# Distinct susceptibility and applicability of MDCK derivatives for influenza virus research

**DOI:** 10.1371/journal.pone.0172299

**Published:** 2017-02-16

**Authors:** Shih-Chao Lin, Matthew A. Kappes, Mei-Chun Chen, Chi-Chen Lin, Tony T. Wang

**Affiliations:** 1 Program in Medical Biotechnology, National Chung Hsing University, Taichung, Taiwan; 2 Center for Infectious Diseases, SRI International, Harrisonburg, Virginia, United States of America; 3 Department of Biomedical Sciences, National Chung Hsing University, Taichung, Taiwan; 4 Department of Biotechnology, Asia University, Taichung, Taiwan; 5 Department of Medical Research, China Medical University Hospital, Taichung, Taiwan; Icahn School of Medicine at Mount Sinai, UNITED STATES

## Abstract

Madin-Darby Canine Kidney (MDCK) cells are widely utilized as a substrate for influenza virus isolation and propagation due to the high yields of virus. Here we compared the conventional MDCK cell line, MDCK-SIAT1 and MDCK-London for viral production, cell survival, and suitability in testing antivirals using six influenza strains including two H1N1 (pandemic and epidemic strains), three H3N2 and one influenza B strain. Overall our results suggest that MDCK-London cell line is superior for virus culturing and quantification, and hence an ideal platform to evaluate antiviral drug efficacy against multiple strains of influenza. Our data also suggests that while virus titers determined by the hemagglutination assay (HA) and neuraminidase activity (NA) are widely used to indicate viral load, there is a poor correlation between these measurements and the infectious titer obtained by plaque assay.

## Introduction

Continued circulation of endemic and pandemic isolates of influenza A and B virus pose a major threat to global public health. Several cell systems have been developed to support the study of the biology of influenza virus and the development of therapeutics, such as the A549 (human lung epithelial cells) [1, 2], Mv 1 Lu (mink lung epithelial cells)[3], LLC-MK2 (Rhesus monkey kidney epithelial cells [2, 4], Vero (African green monkey kidney epithelial cells) [5] and the Madin and Darby Canine Kidney (MDCK) system. The MDCK cell line was established in 1958 by S. H. Madin and N. B. Darby from the kidney tissue of an adult female cocker spaniel [6] and several MDCK derivatives are widely used to study influenza *in vitro* [7]. For example, MDCK-SIAT1 was developed to over-express α-2,6 lineage sialyltransferase in 2003 and reportedly displayed better sensitivity to neuraminidase inhibitors [8]. Independent studies, however, have shown that MDCK-lineage cells exhibit varied susceptibilities to influenza [9]. It was shown that influenza A and B strains propagated from MDCK-SIAT1 cells appeared to have stronger abilities to agglutinate red blood cells (RBCs) and may be a superior substrate for isolation or propagation of clinical influenza specimen [10]. Another MDCK derivative, the MDCK-London clone, showed greater sensitivity to influenza than the parental line [11, 12]. In the present study, we sought to evaluate the applicability of different MDCK cell lines for influenza virus research by measuring viral growth, HA and NA titers, or suitability in testing efficacy of antivirals. Overall our results suggest that the MDCK-London cell line is an ideal platform to influenza virus research.

## Materials and methods

### Cells and viruses

MDCK, MDCK-SIAT1, MDCK-London (FR-58) and all six influenza strains tested in this study, were obtained through the American Type Culture Collection (ATCC, Manassas, VA USA), International Reagent Resource (IRR formerly Influenza Reagent Resource), and Centers for Disease Control and Prevention (CDC, Atlanta, GA USA). All six of the influenza strains were propagated from embryonated chicken eggs before conducting following assays. The H1N1 strains used in this study are the epidemic A/Brisbane/59/2007 strain and the pandemic A/New York/18/2009 strain. The three H3N2 strains are A/Brisbane/10/2007, A/Uruguay/716/2007, and A/Wisconsin/67/2005; the influenza B strain used in this study is B/Jiangsu/361/2002.

### Viral infection

Cells were seeded in 25T flasks 24 hours before infection. Cells were infected at a M.O.I. of 0.01 by inoculating at 0.08 mL per cm^2^ at 37°C for 1 hr to allow viral internalization followed by adding 5 mL DMEM containing 2 μg/mL TPCK-trypsin. At 24, 48, 72 and 96 hours post infection, supernatant was collected and centrifuged at 800 rcf for 10 mins at 4°C. Single use clarified supernatant aliquots were stored at -80°C for downstream analysis.

### Immunostaining plaque assay

Viral samples from supernatant of infected MDCK-lineage cells were 10-fold serially diluted with MEM and used to inoculate 90% confluent monolayer MDCK-London cells for 1 hr at 37°C. Equal volumes of 2.5% Avicel diluted in 2X MEM was added to the inoculum to yield 1.25% Avicel, 1X MEM final concentration. Plaque assays were incubated for 24 hours followed by immunostaining protocol. In brief, at room temperature, cells were fixed with 4% paraformaldehyde for 1hr, permeabilized with 0.05% Triton X-100 PBS buffer for 15mins, and stained with 1:1000 dilution of anti-flu NP or anti-influenza B primary antibody (Stock concentrations are 1 mg/mL and 2.5 mg/mL, respectively, MBS^®^, USA) followed by adding a 1:2000 dilution of anti-mouse secondary antibody (Stock concentration 400 μg/mL, Santa Cruz^®^, USA). Plates were washed (0.05% Tween-20/PBS) 3 times after each step. Colorimetric staining was completed per manufacturer’s instruction using the True Blue reagent (KPL^®^, USA).

### Neuraminidase assay

Commercial neuraminidase assay kit (Thermofisher, USA) was used per manufacturer’s instructions to determine the NA titers. Briefly, equal volume of virus supernatant and neuraminidase substrate were mixed together for 30 mins in white optical 96-well plates (Corning^®^, USA) at ambient temperature. After 30 min incubation, 60 μL of accelerator was added to each well and the luciferase activity in plates was read using a microplate luminometer (Veritas^®^, USA).

### Hemagglutinin titration

Hemagglutinin titration was modified from the method of Fazekas de St. Groth and Grahma [13] and performed in V-bottom 96-well microplates (Corning^®^, USA). 50 μL of PBS was added to each well of microplates followed by adding equal volumes of 2-fold diluted viral samples. Then turkey red blood cells in Alsever (Lampire^®^, USA) were added to all wells. Samples were incubated for at least one hour to allow the RBC agglutination prior to reading the results.

### Neutral Red Uptake (NRU) assay

Neutral red dye uptake assay was used to evaluate and quantify the cell survival rate [14]. Briefly, cells were pre-treated with antiviral drugs either Oseltamivir carboxylate (USP^®^, USA) or Ribavirn (Sigma^®^, USA) for 30 mins. Pretreated monolayers were inoculated with virus at 0.01 M.O.I. and infection progress lasted for 48 to 72 hours until viral control groups reach to at least 90% of cytopathic effects (CPE). The supernatant were removed and 100 μL of MEM containing 0.01% (w/v) neutral red was added to each well of microplates and incubated in the37°C, 5% CO2 incubator for 2 hours. Neutral red/MEM was then removed and plates were washed twice with PBS followed by extracting the dye with Sorensen Citrate Buffer and gently rocked the plates for 5 mins. Lastly, the color intensity of neutral red was determined by a ELISA reader (SpectraMax Plus 385, Molecular Devices^®^, USA) at a wavelength of 540nm.

### MTS assay

MTS (3-(4,5-dimethylthiazol-2-yl)-5-(3-carboxymethoxyphenyl)-2-(4-sulfophenyl)-2H-tetrazolium)) assay is a colorimetric assay to determine the mitochondrial metabolic rate by quantifying the reduced formazan product of MTS in cells to reflect viable cells [15, 16]. MTS assay in this study is accordance with a protocol provided by manufacturer (Promega, Madison, WI USA). In brief, MDCK-lineage cells were seeded in 96-well plates overnight prior to be infected by various influenza viral strains with a M.O.I. of 0.01. One-fifth volume of MTS reagent was added into the culture supernatant at indicated time points. Plates were incubated at 37°C for 1 hour with 5% CO_2_ followed by measuring the absorbance at 490nm with a ELISA reader (SpectraMax Plus 385, Molecular Devices, USA).

### Lectin staining for sialic acid

Sialic acids are a family of oligosaccharide chains on glycoproteins and glycolipids of cellular surfaces which are recognized by influenza viral lectin hemagglutinin [17]. We used two types of fluorescein-conjugated lectins, Sambucus Nigra Lectin (SNA) and Maackia Amurensis Lectin II (MAL II) to stain MDCK-lineage cells for investigating the expression level and distribution of sialic acids. SNA lectins appear to prefer to bind sialic acid with α-2,6 lineage structure, while MAL II preferred an α-2,3 lineage structure [17]. Cells were seeded on 8-well chamber slides (Lab-tek^®^, USA) overnight before treatment with 5% BSA for 30mins in the 37°C incubator followed by incubating with 1:200 dilution of MAL II and SNA lectins conjugate fluorescein (Vector^®^, USA) at 37°C incubator for 1 hour. DAPI was added into chamber wells for 5 mins and washed 3 times with PBS. Slides were inspected by using confocal microscope (LSM-700, Zeiss, Germany). The fluorescent intensity was then quantified by software ImageJ (Version 1.50i, National Institute Health, Bethesda, MD USA) following the measuring procedures provided by Dr. Burgress [18].

### Statistical analysis

All experiments were performed at least three times as indicated in the figure legends. Except were specified, bar graphs were plotted to show mean ± standard deviation (SD). Statistical analyses were performed using Prism 5. A p-value of <0.05 in the Student's test was considered statistically significant.

## Results and discussion

### Distinct ability of MDCK derivatives in amplifying influenza virus strains

We chose six influenza virus isolates including two H1N1 strains, A/Brisbane/59/2007 and A/New York/18/2009, three H3N2 strains, A/Brisbane/10/2007, A/Uruguay/716/2007, and A/Wisconsin/67/2005, and one Influenza B strain, B/Jiangsu/361/2002, and three MDCK cell lines including the conventional MDCK cells, MDCK-SIAT1, and MDCK-London cells to investigate the susceptibility and applicability of host cells in influenza study.

**Fig 1:**
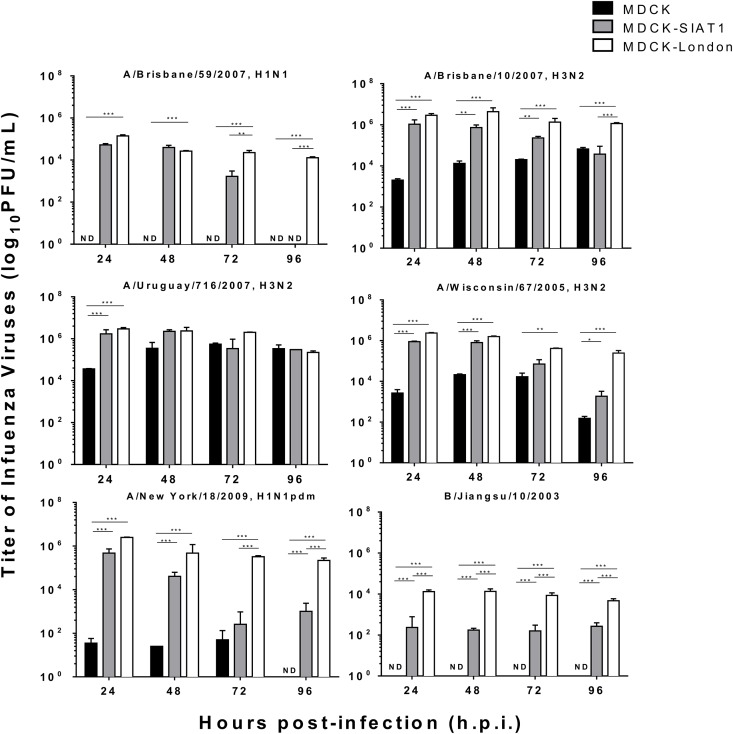
Differential virus growth kinetics in three MDCK cell lines. MDCK-lineage cell lines were infected βy epidemic H1N1 (A), pandemic H1N1 (B), H3N2 (C-E), and influenza B strain (F) with a M.O.I. of 0.01. Supernatant were collected at 24, 48, 72, and 96 hours post-infection. Viral titers were determined by Immunostaining Plaque Assay with MDCK-London cells. N.D. indicates Not Detectable. Data expressed as means ± SEM of three experiments in each group. *p <0.05, **P<0.01, ***p<0.001.

First, conventional MDCK, MDCK-SIAT1, and MDCK-London cells were infected with the above six influenza virus strains and determined infectious titers in the supernatants at 24, 48, 72, and 96 hours post-infection (h.p.i.) using standard plaque assay. The infectious titers (PFU/mL) peaked between 24–48 h.p.i. in supernatants derived from both MDCK-London and MDCK-SIAT1 cell lines. By contrast, conventional MDCK cells yielded viruses with either much lower infectious titers or not infectious at all in the case of A/Brisbane/59/2007 (H1N1 strain) and B/Jiangsu/10/2003 (Fig 1).

### Discordance between plaque assay and HA assay

To corroborate the above results, we performed hemagglutination assay (HA assay) using the same supernatants derived from the above experiments. HA assay measures the ability of influenza A and B viruses to agglutinate avian erythrocytes [19] and is commonly used as a rapid and cost-effective method to determine influenza titers. The HA titers, defined as hemagglutinin unit (h.a.u.), along with the corresponding infectious titers in PFU/ml of multiple influenza strains are summarized in Table 1. Overall the HA titers of the six influenza virus strains increased in a time-dependent fashion, and supernatants derived from MDCK-London and MDCK-SIAT1 cells yielded viruses with higher HA titers than that from conventional MDCK cells. However, the HA titers were not well correlated with the infectious titers (PFU/ml). To our surprise, the HA titers of conventional MDCK cells-derived viruses were not detectable in this study although some of the samples clearly contained infectious viruses (Table 1 and Fig 1). We suspect the imperfect correlation between the determined HA titers and infectious titers was either due to the limited dynamic range or sensitivity of the HA assay.

**Table 1:** Hemagglutination (h.a.u.) and infectious viral titers (Log_10_ PFU/mL) of influenza strains in MDCK-lineage cell lines. HA titers were determined by hemagglutinin activities of influenza to inhibit the agglutination of red blood cells. Supernatant were collected after multiple influenza infections at 24, 48, 72, and 96 h.p.i. Serially diluted viral samples from supernatant were mixed with turkey red blood cells to perform HA titration.

**A. MDCK-London**	24		48		72		96 hpi	
	HAU*	PFU^#^	HAU	PFU	HAU	PFU	HAU	PFU
A/New York/18/2009	1:32	6.4	1:128	5.7	1:128	5.5	1:128	5.4
A/Brisbane/59/2007	1:64	5.2	1:256	4.4	1:512	4.4	1:512	4.1
A/Wisconsin/67/2005	1:32	6.4	1:128	6.2	1:128	5.6	1:128	5.5
A/Uruguay/716/2007	1:32	6.5	1:64	6.4	1:64	6.3	1:64	5.3
A/Brisbane/10/2007	1:8	6.4	1:16	6.6	1:16	6	1:8	6.1
B/Jiangsu/10/2003	1:2	4.1	1:64	4.1	1:64	3.9	1:64	3.7
**B. MDCK-SIAT1**	HAU	PFU	HAU	PFU	HAU	PFU	HAU	PFU
A/New York/18/2009	1:4	5.7	1:16	4.6	1:16	2.4	1:16	3.0
A/Brisbane/59/2007	1:32	4.7	1:64	4.6	1:64	3.1	1:64	ND
A/Wisconsin/67/2005	1:4	5.9	1:16	5.9	1:16	4.8	1:16	3.3
A/Uruguay/716/2007	1:32	6.2	1:64	6.4	1:64	5.5	1:64	5.5
A/Brisbane/10/2007	1:4	6.0	1:8	5.9	1:8	5.4	1:16	4.8
B/Jiangsu/10/2003	<1:2	2.4	1:2	2.2	1:2	2.2	<1:2	2.4
**C. MDCK**	HAU	PFU	HAU	PFU	HAU	PFU	HAU	PFU
A/New York/18/2009	<1:2	1.5	<1:2	1.4	<1:2	1.7	<1:2	ND
A/Brisbane/59/2007	<1:2	ND	<1:2	ND	<1:2	ND	<1:2	ND
A/Wisconsin/67/2005	<1:2	3.4	<1:2	4.3	<1:2	4.2	<1:2	2.2
A/Uruguay/716/2007	<1:2	4.6	<1:2	4.6	<1:2	5.7	<1:2	5.5
A/Brisbane/10/2007	<1:2	3.3	<1:2	4.2	<1:2	4.3	<1:2	4.8
B/Jiangsu/10/2003	<1:2	ND	<1:2	ND	<1:2	ND	<1:2	ND

*HAU: Hemagglutinon Unit; PFU: Plaque-forming Unit; hpi: hours post-infection;

^#^PFU titer is expressed as log_10_ PFU/mL.

Subsequently we measured the neuraminidase (NA) activity in the same supernatants derived from the above study. NA activity is another readout for rapid quantification of influenza virus [20]. Shown in S1 Fig, the NA activity scores of most tested influenza strains propagated on MDCK-London and MDCK-SIAT1 cells were consistently higher as opposed to those from conventional MDCK cells, reinforcing the observation that MDCK-London and MDCK-SIAT1 cells yielded more viruses than did the conventional MDCK cells. Notably, the NA titer exhibited similar trend pattern and is more accordance with infectious titer determined by plaque assay comparing to HA titer.

### Cytopathic effect on MDCK cells

**Fig 2:**
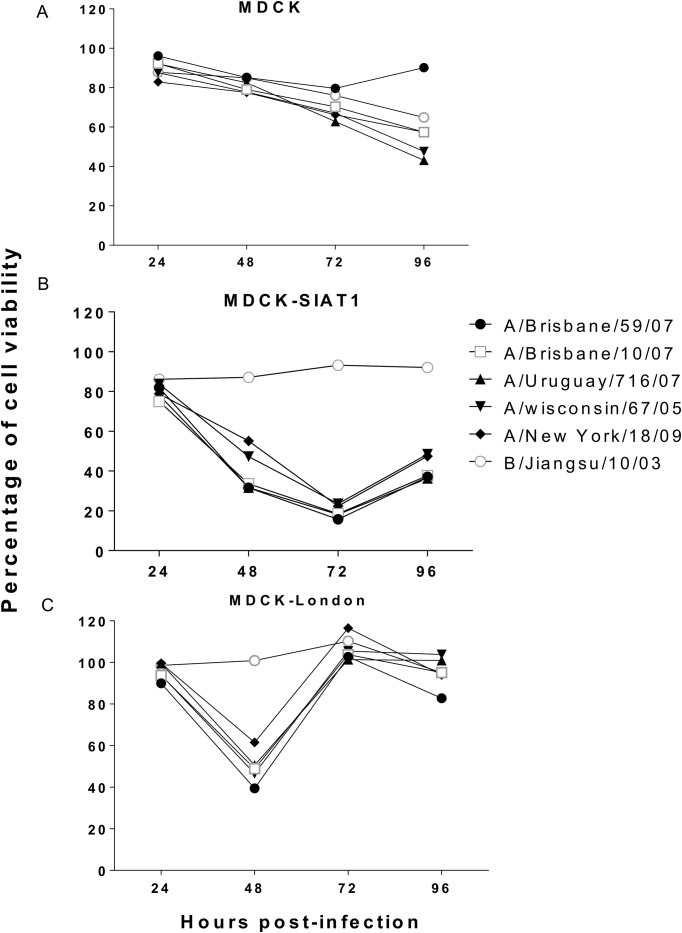
Cell viabilities of virus infected MDCK cells. Cellular viability was determined as the concentration of formazan product level. MTS tetrazolium compound was added into influenza-infected MDCK (A), MDCK-SIAT1 (B), and MDCK-London (C) at 24, 48, 72, and 96 hours post-infection followed by incubating at 37°C for 1 hour. Concentration of formazan product was quantified with ELISA reader at the wavelength 490 nm. The baseline of viability from mock-infected cells (media only, no virus) was set to 100%. Data expressed as means ± SEM of three experiments in each group.

**Fig 3:**
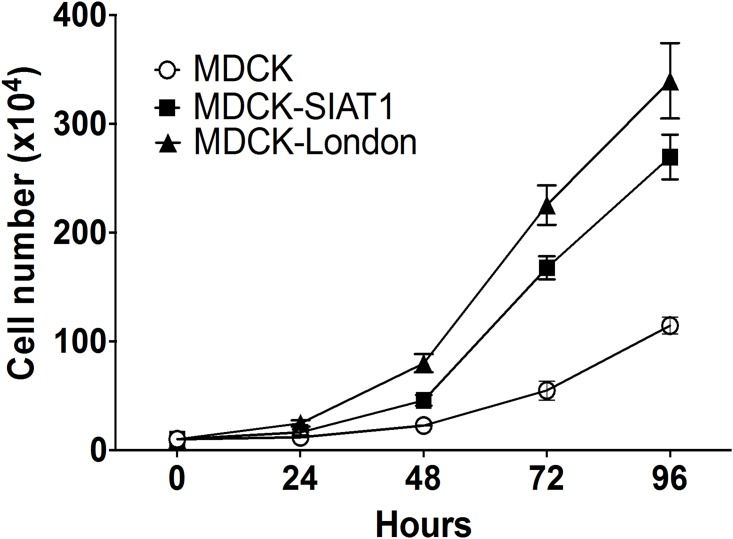
Cellular growth rates of MDCK-lineage cells. Cells were seeded in plates with 10% FBS complete DMDM for 24 hours before counting numbers to allow adherence on the plates. The numbers of each three MDCK-lineage cells were counted by hemacytometer for at least 3 times in each independent experiment.

The cytopathic effect (CPE) associated with influenza virus infection may be an useful indicator of peaked virus production [21]. Measurement of tetrazolium formazan products by MTS or MTT assay is frequently used for evaluation of the level of cell viability [22, 23]. Here we performed MTS assay to investigate whether the cellular viability correlated with viral titers. As expected, the cellular viabilities of all cell lines except conventional MDCK cells drastically decreased after 24 hours infection (Fig 2). While the viability of infected conventional MDCK cells continued to decrease during the MTS experiments, the viability of MDCK-SIAT1 cells dropped to the lowest point at 72 h.p.i. and that of MDCK-London cells at 48 h.p.i.. Of note, the cell viability of MDCK-SIAT1 and MDCK-London rebounded by 72 and 48 h.p.i., respectively, which may be due to the robust growth of these two lines. Indeed, MDCK-London and MDCK-SIAT1 cells displayed faster growth rates than conventional MDCK cells (Fig 3). The trends of cellular viabilities, however, do not correlate with that of the PFU titers (Fig 1), suggesting the cellular viability may not be a quality indicator of infectious titers.

### Applicability of MDCK Derivatives in antiviral testing

Next, we tested if antiviral drugs exhibit similar efficacy using these three cell systems. Two antiviral drugs, oseltamivir carboxylate and ribavirin, were added to cells along with viral infections and the 50% effective concentrations (EC50) were determined (Table 2). To our surprise, while the nucleoside analogue ribavirin displayed varied efficacy against different influenza virus strains on three lines, the neuraminidase inhibitor, oseltamivir carboxylate, which is supposed to show improved efficacy on MDCK-SIAT1 cells, only protected MDCK-SIAT1 and MDCK-London cells from A/New York/18/2009 and A/Brisbane/59/2007 infections, suggesting the antiviral efficacy of oseltamivir carboxylate may be strain-specific.

**Table 2:** Varied efficacies of antiviral drugs on different MDCK-lineage cells. MDCK, MDCK-SIAT1, and MDCK-London were pretreated with two antiviral drugs, Oseltamivir carboxylate and Ribavirin, before Influenza infections. The concentrations of ribavirin and oseltamivir carboxylate started with 100 μg/mL followed by 2-fold serial dilution. Neutral red uptake assays were performed to evaluate the degree of cellular survival and were quantified by ELISA reader at wavelength 540 nm.

Unit of EC_50_: μg/mL*		A/New York/18/09	A/Uruguay/716/07	A/Brisbane/10/07	A/Brisbane/59/07	A/Wisconsin/67/05	B/Jiangsu/10/03
MDCK	Ribavirin	68.5	29.3	91.4	24.5	67.4	>100
	Oseltamivir	42.5	31.5	78	30.6	17.1	39.4
MDCK-SIAT1	Ribavirin	8.9	21.3	25.4	13.5	25.4	>100
	Oseltamivir	1.7	>100	>100	32.9	>100	35.5
MDCK-London	Ribavirin	13.1	13.2	31.3	19.3	34.4	18
	Oseltamivir	2.89	>100	>100	1.34	>100	>100

*Oseltamivir carboxylate and ribavirin displayed cytotoxicity at concentrations above 80 and 400 μg/mL, respectively, on all MDCK-lineage cells.

**Fig 4:**
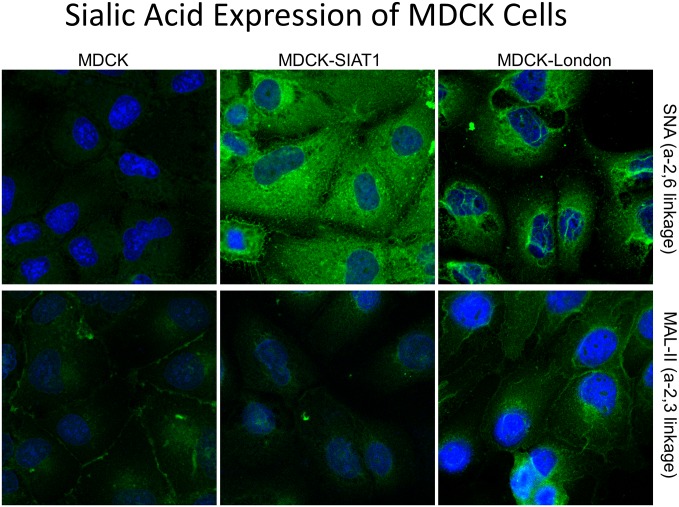
The expression and distribution of sialic acids in three MDCK-lineage cell lines. Cells were seeded on chamber slides and stained with *Sambucus nigra* agglutinin (SNA, A-C) and *Maackia amurensis* agglutinin (MAL-II, D-F) lectins conjugated fluorescein after BSA blocking. Images were taken by confocal microscopy in a 680×magnification. Green fluorescence indicates the location of sialic acids and blue fluorescence stained by DAPI showed the nucleus of cells. Corrected total cell fluorescence (CTCF) was determined with software Image J (version 1.50i, Bethesda, MD USA). The highest density among three cell lines was defined as “high” whereas weakest was defined as “low”; density between high and low was considered “moderate”.

To probe into the mechanism of the observed heterogeneity of the MDCK cell lines, we examined the sialic acid (SA) expression and distribution because expression of SA, a cellular receptor of Influenza [24], is known to control the cellular susceptibilities to influenza virus infection. To probe SA expression and localization, SA-galactose (SA-Gal) specific lectins *Maackia amurensis* agglutinin (MAL-II; SAα-2,3Gal specific) and *Sambucus nigra* agglutinin (SNA; SAα-2,6Gal specific) were used [25]. It was noticed that MDCK-SIAT1 expressed higher levels of α-2,6 linked of sialic acids but not α-2,3 linkage of sialic acids, which confirms a previous report [8]. Meanwhile, MDCK-London cells expressed both α-2,6Gal and α-2,3Gal sialic acids at a moderate level, whereas conventional MDCKs showed relatively low levels of sialic acid expressions of both SA structures (Fig 4 and S2 Fig).

## Discussion and conclusion

Understanding the heterogeneity of the MDCK cell line and its derivatives is critical to their applicability for influenza virus research. HA, NA, and plaque assays are foundational tools used to detect and quantify influenza virus within diagnostic and research settings. Our findings suggest that cautions must be taken when interpreting viral titers measured by different assays on different MDCK cell lines, as there is only a poor correlation between these measures. Although HA and NA titers may not accurately reflect the peak yield of the virus, these assays can be useful for rapid detection and rough quantification of HA or NA respectively. Surprisingly, low and no viral titers of most influenza strains were exhibited in conventional MDCK cells which might require further optimization of the culture conditions (temperature or inoculation period) for influenza propagations in this MDCK cell line.

MTS assay has been widely utilized to measure cell viability or more precisely, mitochondrial metabolic rate. We measured the cell viability of different MDCK-lineage cells after infection by various influenza strains (Fig 2). Within the first 48 hour after infection, the cellular metabolic rates of the three cell lines all decreased. Interestingly, the metabolic rate of MDCK-London recovered to the normal level by 72 h.p.i., which was reflected by the increase in formazan product levels. Noteworthy, the cell growth rate of MDCK-London cells is faster in comparison to the other two MDCK-lineage cells (Fig 3). Not surprisingly, the cellular metabolic rate of MDCK-London is highest among these cell lines. Despite the CPE caused by influenza virus infection, leftover MDCK-London cells were able to quickly proliferate and recover in numbers, which explains the dip of cell viability of MDCK-London cells at 48 hours and then the recovery of viable rate thereafter (shown in Fig 2).

To our knowledge, there are only a few reports where the MDCK-London cell line was utilized as substrates for influenza. [12, 26] and the characteristics of MDCK-London cells for such studies are limited. Therefore, we evaluated the applicability of MDCK-London for all influenza strains tested in this study and our data revealed that influenza virus propagated well in MDCK-London cells, making them an ideal platform for drug testing. The susceptibility of MDCK-London is likely due to the expression of both α-2,6 and α-2,3 linked of sialic acids, which renders the cells susceptible to all tested influenza virus strains. Given the type of sialic acid on MDCK cells likely plays a crucial role in propagation/detection of clinical influenza isolates, MDCK-London cells appear to be a superior substrate for influenza virus research.

## Supporting information

S1 Fig Neuraminidase titer changes after influenza infection. Neuraminidase activities of six influenza strains, including H1N1, H3N2, and influenza B, were monitored after viral infection at 24, 48, 72, 96 hours. Viral supernatant were mixed with substrate of neuraminidase and accelerator and completed per manufacturer’s instruction as mentioned in materials and method. Data expressed as means ± SD and the data presented are representatives of three independent experiments with similar results.
(TIF)

S2 Fig Quantification of fluorescent density of lectin staining. Corrected total cell fluorescence (CTCF) was analyzed from three distinct cells in each cell line of indicated lectin staining by software Image J v1.50i followed by calculating with the formula: Integrated Density–(Area of selected cell X Mean fluorescence of background readings).
(TIF)
