# Supplementary figures and images for: Distinct susceptibility and applicability of MDCK derivatives for influenza virus research
Source: PLoS One. 2017 Feb 16;12(2):e0172299. doi: 10.1371/journal.pone.0172299 (PMC5313193; doi:10.1371/journal.pone.0172299)

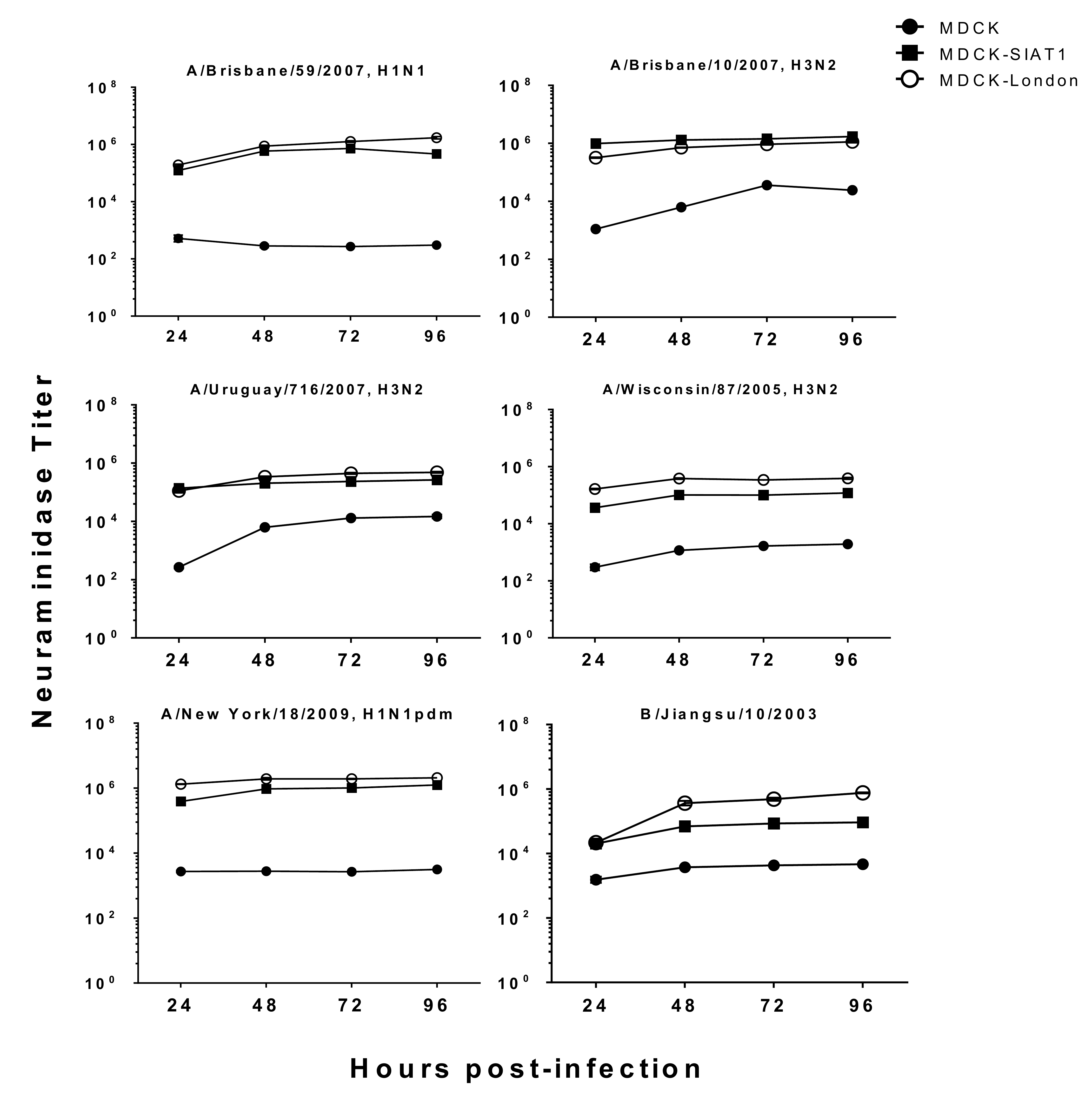

Supplement: S1 Fig — Neuraminidase activities of six influenza strains, including H1N1, H3N2, and influenza B, were monitored after viral infection at 24, 48, 72, 96 hours. Viral supernatant were mixed with substrate of neuraminidase and accelerator and completed per manufacturer’s instruction as mentioned in materials and method. Data expressed as means ± SD and the data presented are representatives of three independent experiments with similar results. (TIF) [file pone.0172299.s001.tif]

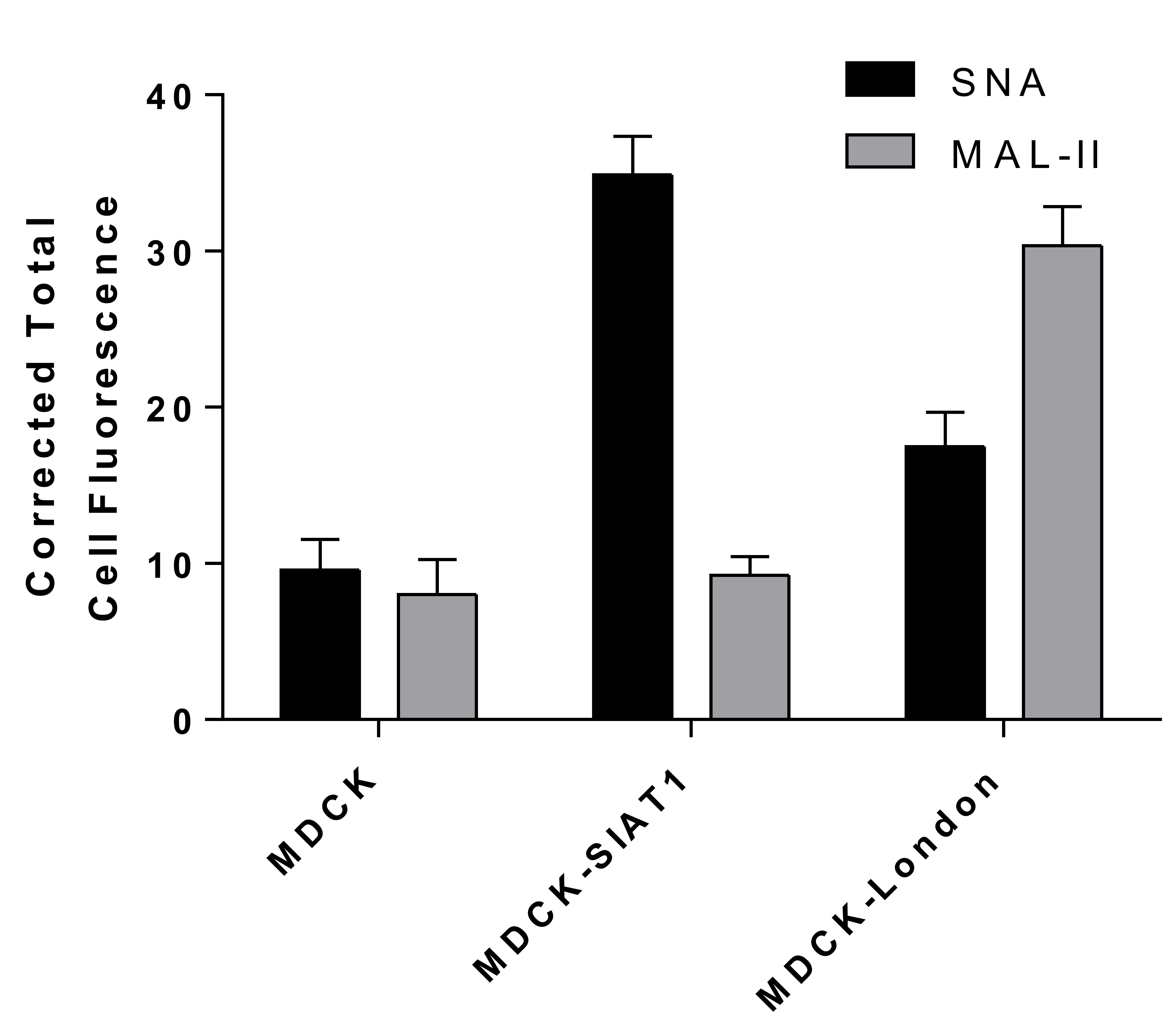

Supplement: S2 Fig — Corrected total cell fluorescence (CTCF) was analyzed from three distinct cells in each cell line of indicated lectin staining by software Image J v1.50i followed by calculating with the formula: Integrated Density–(Area of selected cell X Mean fluorescence of background readings). (TIF) [file pone.0172299.s002.tif]
